# CAS Common Chemistry in 2021: Expanding Access to Trusted Chemical Information for the Scientific Community

**DOI:** 10.1021/acs.jcim.2c00268

**Published:** 2022-05-13

**Authors:** Andrea Jacobs, Dustin Williams, Katherine Hickey, Nathan Patrick, Antony J. Williams, Stuart Chalk, Leah McEwen, Egon Willighagen, Martin Walker, Evan Bolton, Gabriel Sinclair, Adam Sanford

**Affiliations:** † CAS, 2540 Olentangy River Rd, Columbus, Ohio 43202, United States; ‡ Center for Computational Toxicology and Exposure, Office of Research and Development, U.S. Environmental Protection Agency (U.S. EPA), Research Triangle Park, North Carolina 27711, United States; § Department of Chemistry, University of North Florida, Jacksonville, Florida 32224, United States; ∥ Physical Sciences Library, Cornell University, Ithaca, New York 14853, United States; ⊥ Department of Bioinformatics - BiGCaT, Maastricht University, 6229 ER Maastricht, The Netherlands; # Department of Chemistry, SUNY Potsdam, 44 Pierrepont Ave., Potsdam, New York 13676, United States; ∇ Department of Health and Human Services, National Center for Biotechnology Information, National Library of Medicine, National Institutes of Health, 8600 Rockville Pike, Bethesda, Maryland 20894, United States

## Abstract

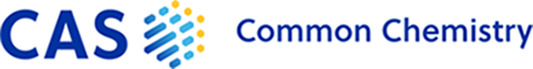

CAS Common Chemistry (https://commonchemistry.cas.org/) is an open web resource that provides access to reliable chemical substance information for the scientific community. Having served millions of visitors since its creation in 2009, the resource was extensively updated in 2021 with significant enhancements. The underlying dataset was expanded from 8000 to 500,000 chemical substances and includes additional associated information, such as basic properties and computer-readable chemical structure information. New use cases are supported with enhanced search capabilities and an integrated application programming interface. Reusable licensing of the content is provided through a Creative Commons Attribution-Non-Commercial (CC-BY-NC 4.0) license allowing other public resources to integrate the data into their systems. This paper provides an overview of the enhancements to data and functionality, discusses the benefits of the contribution to the chemistry community, and summarizes recent progress in leveraging this resource to strengthen other information sources.

## Introduction

CAS, a division of the American Chemical Society, has collected, curated, and analyzed the world’s published science as part of its vision to improve people’s lives through the transforming power of chemistry since 1907.^1,2^ Scientists, manufacturers, regulators, and data scientists around the world rely on CAS for accurate information on chemical substances. CAS Common Chemistry, an open resource based on a subset of chemical substance content from CAS REGISTRY, was first launched in 2009 by CAS to strengthen the accuracy of publicly available scientific information.^3^

**Figure 1:**
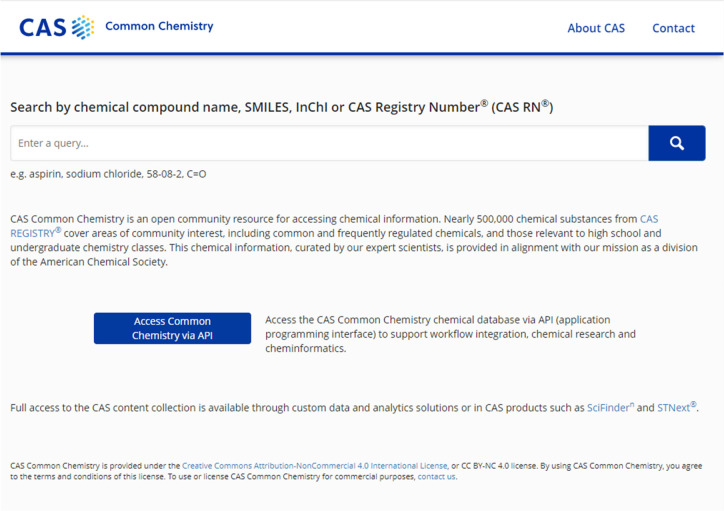
New CAS Common Chemistry homepage.

**Figure 2:**
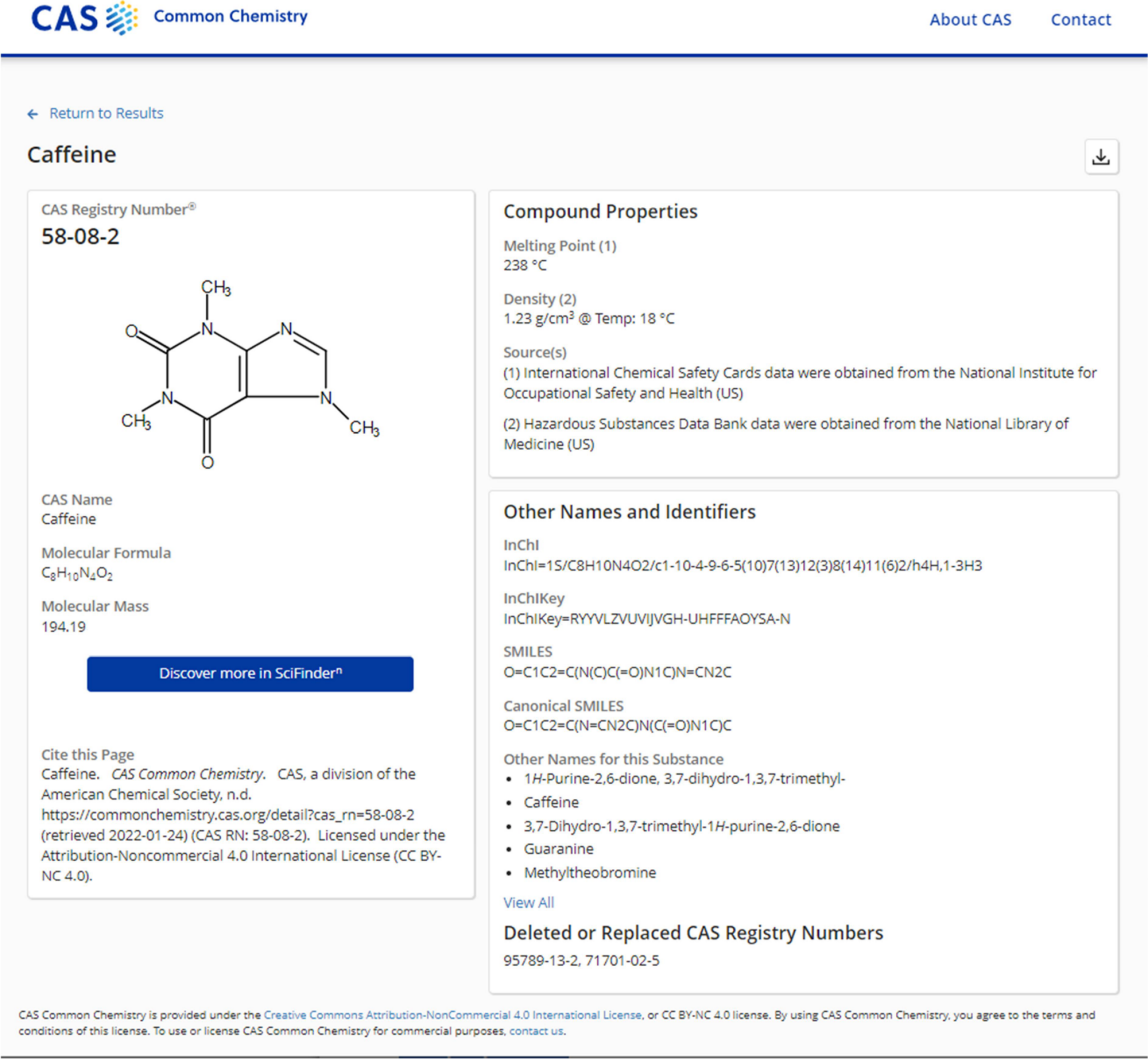
Detailed record for caffeine in CAS Common Chemistry.

CAS Common Chemistry was established to provide a reliable source of chemical identifiers and associated information to the general public as part of the mission of the ACS. It enables millions of visitors to obtain reliable scientific information on nearly 500,000 substances through search or application programming interface (API) functionality. Users leverage this information in a variety of ways, including in teaching and learning, to promote safe practices, and to support research. Additionally, Wikipedia has leveraged the resource since its inception in 2009 to provide accurate CAS Registry Numbers for the most ubiquitous chemical substances.^4^ For each included substance, a substance detail page provides key attributes as well as a citation to support referencing in academic studies. For examples of search results, see substance detail pages for caffeine, aspirin, and benzene, as well as Figures 1 and 2.

## Updates to the CAS Common Chemistry Dataset

The 2021 release has several key enhancements to strengthen the CAS Common Chemistry dataset, including an expanded number of substances, enhanced associated chemical information, validated CAS Registry Numbers, and additional chemical representations.

### Expanded Dataset Sourced from CAS REGISTRY

CAS Common Chemistry contains substances and related data from CAS REGISTRY, the largest scientist-curated chemical substance database in the world, crossing 250 million chemical substances in April 2021 and growing daily.^5,6^ In response to requests from the scientific community, the number of substances openly released in CAS Common Chemistry was recently expanded to nearly 500,000 substances. This is a dramatic increase from the 8000 substances initially available. The collection represents substances and related data for chemicals of concern, consumer product ingredients, commonly regulated chemicals, and chemicals frequently used in undergraduate chemistry curricula.

### Enhanced Associated Chemical Information

**Figure 3:**
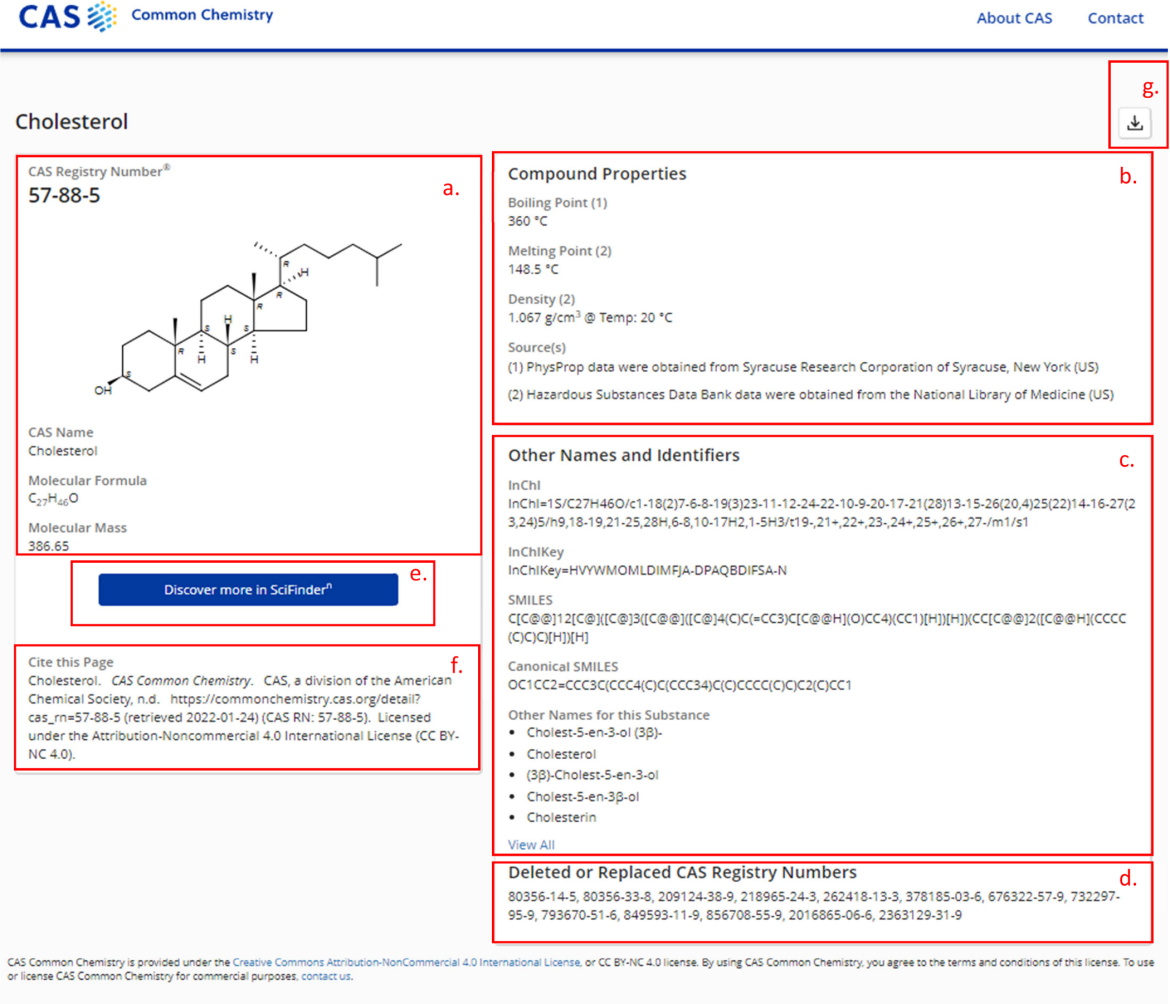
CAS Common Chemistry detail page for cholesterol, with elements as follows: (a) Basic information for the substance that includes chemical name, CAS RN, molecular formula, molecular mass, and structure image. (b) Basic property information that includes boiling point, melting point and density, where available. (c) Other names and identifiers, including InChI, InChIKey, SMILES, canonical SMILES, and synonyms. (d) Deleted or replaced CAS RNs that include any CAS RNs that referred to this substance in the past, but are no longer active. (e) Link to CAS SciFinder^n^ that directly links the user to find more information about the substance. (f) Pre-formatted citation that can be copied and pasted directly to cite this webpage. (g) Download button that downloads the molfile format for this chemical structure.

Each substance in the resource includes its chemical name, chemical structure image, molecular formula, and molecular weight. The resource is further enhanced by chemical synonyms, which may include systematic chemical names, common names, and trade names for each chemical substance. Basic substance properties—boiling point, melting point, and density—are also included where available. This is demonstrated in Figure 3a,b.

For users of CAS SciFinder^n^, a quick link is also included to launch the product and quickly discover more information about the substance, including additional properties, spectral data, literature references, regulatory details, commercial suppliers, and chemical reactions in which it is a participant (Figure 3e).

### Validated CAS Registry Numbers

Each substance is identified by its CAS Registry Number or CAS RN (Figure 3a). This unique identifier, which is assigned by CAS as part of the curation of CAS REGISTRY, is the most common way to identify chemical substances on consumer products, regulatory lists, chemical catalogs, and more. It offers scientists and non-scientists alike a way to disambiguate the complexity of chemical identity quickly and easily. CAS RNs are included in many freely accessible chemistry websites that aggregate data from various sources; however, these CAS RNs are typically not obtained directly from CAS and as such are not validated and can have significant data quality issues.^7−10^ Because CAS is the authoritative source of CAS RNs, the CAS RNs included in CAS Common Chemistry are validated, accurate, and trustworthy.

Occasionally, two CAS-registered substances can be determined to be chemically equivalent – for example, if one of the substances was initially known only by a trade name without a full chemical structure. Any additional CAS RNs to identical substances are also included in CAS Common Chemistry to aid users in resolving these identifiers to their accurate, current counterparts (Figure 3d).

### Additional Chemical Structure Representations

Chemical structure representations enhance the ability to cross-reference substances between resources. The original CAS Common Chemistry resource did not include any computer-readable representations of the chemical structure—only structure images. CAS Common Chemistry now includes several structural representations, including the International Union of Pure and Applied Chemistry (IUPAC) International Chemical Identifier, or InChI, a nonproprietary string representation of the chemical structure, as well as the associated InChIKey, a fixed-length hash string that is intended to enhance the text-based searchability of the InChI chemical structure.^11,12^ Simplified Molecular-Input Line-Entry System or SMILES strings, another string representation of the structure, are also included.^13^ Molfile format is available for download.^14^ See Figure 3c,g for examples.

## Updates to Application and Integration Features

Additional steps were taken in the 2021 update to ensure that the data can be effectively utilized in common use cases. Several new considerations ensure that CAS Common Chemistry is more broadly applicable to the community, including enhanced searchability, API integration, and reusable licensing.

### Enhanced Searchability

This content is searchable by chemical name, InChI, InChIKey, SMILES, and CAS RN. InChI, InChIKey, and SMILES are new features of the site that provide significantly improved search capability. Exact searches are supported. Limited wildcard searching is also supported, using “*” at the end of a search string. To search by InChI and InChIKey, the query must begin with a formatting string, “InChI=” or “InChIKey=.”

### API Integration

Newly added API capabilities support digital workflows and cheminformatics initiatives by allowing programmatic access to the data. The API allows access via three endpoints:

- “Search” accepts CAS RN, SMILES, InChI, InChIKey, or chemical name as input. It returns a list of matching substances, including each substance’s chemical name, CAS RN, and structure image.
- “Detail” allows the retrieval of all available information for a known chemical substance. It accepts CAS RN or substance Uniform Resource Identifier (URI) as input and returns all CAS Common Chemistry record information, including identifiers, synonyms, structures, deleted or replaced CAS RNs, and experimental property data.
- “Export” takes CAS substance URI (found in the detail response) as input. It returns the molfile for the substance in question.

Please refer to the Supporting Information for examples of API outputs, schemas, and examples of use.

### Reusable Licensing

Reusable licensing of the content is now provided through a Creative Commons Attribution-Non-Commercial (CC-BY-NC 4.0) license.^15^ This license allows users to copy, redistribute, and build upon the content in CAS Common Chemistry, as long as this is done for exclusively noncommercial purposes with appropriate attribution to CAS. The standardized terms of this license enable CAS Common Chemistry users to quickly understand its allowable use without needing a legal expert to interpret a license document. Open science initiatives and public, noncommercial information resources are now readily able to leverage CAS Common Chemistry content as a result.

To support reuse and referencing by students and researchers, a preformatted citation is also provided for each substance in the resource (Figure 3f).

## Role of Community Collaboration

Since its launch in 2009, CAS Common Chemistry has evolved into a valuable source of reliable chemical information accessed by millions. The collaboration of stakeholders in the chemistry community has provided the input necessary to create and enhance this resource.

The first collaboration occurred in December 2007, when one of the authors (AJW) initiated a project to curate chemical structures on Wikipedia.^25^ In consultation with Wikipedia user “Walkerma” (author MW on this paper), this author engaged with a team of other chemists on the platform to collaboratively curate the data. Part of the activity included an effort to clean up CAS RNs on Wikipedia pages that were not associated with the substance they represent in CAS REGISTRY.^26^ CAS was enlisted to support this project and agreed to formally collaborate in the effort in 2008. This collaboration ultimately resulted in the development and launch of CAS Common Chemistry in May 2009.

As the popularity of chemical information on the internet grew dramatically in the 2010s, community members noted that an additional collaboration with CAS was increasingly needed to further enhance the accuracy and usability of open chemical information.^16^ In 2019, interested parties in the chemistry community proposed to CAS to expand and enhance CAS Common Chemistry. This proposal propelled CAS to move forward with the group’s idea. Throughout 2020, the group met regularly to develop requirements, select substances, prototype, and test the new CAS Common Chemistry, which was ultimately launched in March 2021 (see Figure 1).

## Impact on Other Data Sources

One of the most significant challenges in cheminformatics is the accurate identification and matching of chemical substances. Many structure representations are available, and each has its limitations. Furthermore, converting between these structure formats is an imperfect science and often results in additional inaccuracies because of the limitations of source structure formats or conversion algorithms. Some chemical structure representations offer the ability to encode identical structures in different ways, further amplifying complexity. Identifiers that do not encode structural characteristics as part of their format, such as the CAS RN, provide an alternative that eliminates the complexity of matching chemical structures. While this can be advantageous, it also creates challenges in ensuring that CAS RNs from nonauthority sources are accurate to the underlying substances that they represent.

To overcome limitations in chemical data exchange, many resources have developed their own curation policies for the validation and acceptance of chemical information. These curation policies typically leverage an internal standard for chemical structure representation and then implement a consistent approach for the acceptance of information that may be attempting to represent the same chemical entity. For example, the CompTox Chemicals dashboard developed by the Center for Computational Toxicology at the U.S. Environmental Protection Agency (EPA) has developed a process that combines automated matching approaches with manual curation to resolve chemical complexity.^17,18^ The resource further scores each one of its entries based on data reliability to communicate the potential for inaccuracy in its information.

The PubChem resource was able to extensively leverage the CAS Common Chemistry contents to identify, validate, correct, and highlight CAS RN identifiers within the limitations and constraints of structure representation noted above.^19^ This included adding CAS Common Chemistry as a data source (https://pubchem.ncbi.nlm.nih.gov/source/24603) with an appropriately permissive data license and the creation of a cross-link to the CAS Common Chemistry website for CAS RN identifiers available therein. As a trusted, authoritative source within PubChem, the CAS Common Chemistry data source helps to indicate validated CAS RN. Structural representations from CAS Common Chemistry, notwithstanding the issues of data exchange, are further utilized by PubChem to help automated data checking and consistency approaches, helping to enable appropriate structural representations for a given CAS RN.

The latest release of CAS Common Chemistry has also supported updates and corrections to CAS RNs in Wikidata and Wikipedia.^20^ InChIKeys were calculated from CAS SMILES using Bacting 0.0.31^21^ with the Chemistry Development Kit 2.7.1^22^ and were matched with content in Wikidata. The CAS RNs were then compared. References to CAS Common Chemistry were added for CAS RNs that matched. Mismatches have been shared with the Wikidata and Wikipedia communities so that they can manually review and correct the misleading entries using CAS Common Chemistry as a reference. Because Wikidata also curates identifiers from other data sources, validated CAS RNs in Wikidata may also be used to cross-reference with other resources. Scripts are provided in the Supporting Information.

A long-standing challenge in such curation approaches has been accurate matching to CAS RNs, which do not directly encode chemical information. With its inclusion of several chemical structure representations for each CAS RN, offered under reusable license terms, the 2021 release of CAS Common Chemistry enables and supports the accuracy of open sources of scientific information, including Wikipedia, Wikidata, PubChem, and the CompTox Chemicals dashboard. Considering that the CAS Common Chemistry data set of about 500,000 substances that are among the most important and common chemicals to chemists, it is a sizable, new, and important authoritative data set for the community.

## Future Work

Several additional features that would enhance CAS Common Chemistry have been identified through the development process. These include a more flexible chemical search—the current search mechanism for chemical structure representations supports exact searching only—in addition to the ability to facet or filter search results. Supporting additional fields in search, such as searching of property values, would aid in the application of CAS Common Chemistry in teaching and learning. Additionally, the inclusion of links to regulatory resources or tags that indicate membership in regulatory lists would enhance the usability of CAS Common Chemistry for environmental health and laboratory safety.

One known challenge in leveraging CAS Common Chemistry as an interchange between information sources stems from the proprietary chemical structure encoding format used to create the underlying authoritative representations of chemical information. As such, chemical structure representations provided on the website have the potential to be flawed because of differences in the features supported by each structure format and because of the potential for inaccuracies in the conversion process. Identifying aspects of chemistry that are different between data representations, finding ways to address these limitations and ensuring that conversion algorithms make best use of all available features within each representation format is an area for continued future work both by CAS and by the chemistry community (such as through the enhancement and standardization of chemical representation approaches, including IUPAC InChI and IUPAC SMILES+ efforts^23,24^). In addition to its internal work on these initiatives, CAS participates actively in community efforts to tackle these challenges, including the InChITrust and the Pistoia Alliance.

## Conclusions

The release of the CAS Common Chemistry collection of nearly 500,000 chemical substances is a very significant contribution of curated data to the community and, because of its open data licensing, is available for reuse by third parties. Access to high-quality data from CAS REGISTRY fosters learning and promotes safety.

As the need for reliable chemical information continues to grow, CAS Common Chemistry will continue to be a valuable resource to promote the dissemination of accurate scientific information. Collaboration with the community will ensure that CAS Common Chemistry continues to meet the evolving needs of the global community.

## Data and Software Availability

The solution and content described herein are openly available for noncommercial use at https://commonchemistry.cas.org. In addition to this user interface, the Supplemental Information includes examples for accessing content programmatically via API.

## Supporting Information Available

API outputs, schemas, and examples of use in Java and Python (ZIP)
Scripts demonstrating comparison between CAS Common Chemistry, Wikipedia, Wikidata and HMDB (ZIP)
Examples of CAS Common Chemistry API use from the EPA (PDF)
