# Supplementary material for: CAS Common Chemistry in 2021: Expanding Access to Trusted Chemical Information for the Scientific Community
Source: J Chem Inf Model. 2022 May 13;62(11):2737–43. doi: 10.1021/acs.jcim.2c00268 (PMC9199008; doi:10.1021/acs.jcim.2c00268)
Supplement: Supplementary file 3 — ci2c00268_si_003.pdf [file ci2c00268_si_003.pdf]

Supporting Information

CAS Common Chemistry API Use – Details and Application

The CAS Common Chemistry website provides an application programming interface (API) to enable automated data access by software applications. The API allows a software application to query and utilize the same raw data that is served to human users through the user interface. This data may also be viewed by human users in JavaScript object notation (JSON) format.

Developers may request API access for CAS Common Chemistry using an online form [1].

The CAS Common Chemistry API has three "endpoints," or types of basic functionality:

Search: Access one or more search results by CAS RN, SMILES, InChI, InChIKey, or name (with * as a wildcard character). Equivalent to user search in the homepage search bar. Results include CAS RN, name, and scalable vector graphic (SVG) image.

Example #1: https://commonchemistry.cas.org/api/search?q=methyl%20isocyanate

Example #2: https://commonchemistry.cas.org/api/search?q=methyl*

Example #3: https://commonchemistry.cas.org/api/search?q=InChIKey=HAMGRBXTJNITHG-UHFFFAOYSA-N

Detail: Access one detailed record by CAS RN or uniform resource identifier (URI). Equivalent to user navigating to a single result page from search. Record includes URI, CAS RN, name, SVG image, InChI, InChIKey, SMILES, canonical SMILES, molecular formula, molecular mass, MOL file availability, experimental property values, and citations for experimental property values.

Example #1: https://commonchemistry.cas.org/api/detail?cas_rn=624-83-9

Example #2: https://commonchemistry.cas.org/api/detail?uri=substance/pt/624839

Export: Download MOL file for a record by URI. Equivalent to user clicking structure download button on a single result page.

Example: https://commonchemistry.cas.org/api/export?uri=substance/pt/624839 (clicking will begin a file download)

API Usage Within EPA

The CAS Common Chemistry API serves as a resource for expedient, automated exchange of structure and identifier information based on a highly reliable source. This can be used to validate information from other public sources or to gather information not otherwise available.

For instance, the results of any search query on the PubChem database—for instance, a list of compounds containing a SMARTS substructure or a list of compounds belonging to a classification [2]—can easily be exported from the PubChem user interface. However, the exported data do not include CAS RNs. Using the CAS Common Chemistry API, names or structural identifiers from these data can be

efficiently checked for an associated CAS RN, and these mappings evaluated by human curation teams for use.

In another use case, Markush chemical structures are “enumerated” using a ChemAxon Marvin plugin [3]. This enumeration process generates all potential child structures meeting the Markush definition. These structures, in InChI, InChIKey, or SMILES format, can be searched through the CAS Common Chemistry API to determine whether they have been previously assigned names and CAS RNs. Collating this information programmatically, rather than conducting the entire process by human research, enables human curators to make a final determination of correctness within a smoother and more efficient integrated workflow.

References

1. https://www.cas.org/services/commonchemistry-api
2. https://pubchem.ncbi.nlm.nih.gov/classification/#hid=72
3. https://apidocs.chemaxon.com/marvin/help/developer/beans/api/chemaxon/marvin/calculations/MarkushEnumerationPlugin.html
